# The thinker: opposing directionality of lighting bias within sculptural artwork

**DOI:** 10.3389/fnhum.2015.00251

**Published:** 2015-05-06

**Authors:** Jennifer R. Sedgewick, Bradley Weiers, Aaron Stewart, Lorin J. Elias

**Affiliations:** ^1^Department of Psychology, University of SaskatchewanSaskatoon, SK, Canada; ^2^Department of Computer Science, University of SaskatchewanSaskatoon, SK, Canada

**Keywords:** neuroaesthetics, lighting, sculpture, pseudoneglect, preference, perceived illumination, spatial attention

## Abstract

Individuals tend to perceive the direction of light to come from above and slightly from the left; it has been speculated that this phenomenon is also producing similar lighting preferences within 2-dimensional artworks (e.g., paintings, advertisements). The purpose of the present study was to address if lighting bias was present in the 3-dimensional medium of sculpture by implementing a virtual art gallery lighting paradigm. Thirty-nine participants completed a computer task that consisted of 48 galleries each containing one sculpture (24 original sculptures, 24 mirror-reversed) which was surrounded by eight lights (above/below, left/right, front/back). Participants would select one light source to illuminate the sculpture in a manner they perceived to be the most aesthetically pleasing. The results indicated a significant preference for lights positioned from above and from the right, a finding that is contradictory to previous lighting bias research examining artworks. An interpretation for the rightward bias applies the perceptual concept of subjective lighting equality. Objects illuminated from the left typically appear brighter in comparison to right-side lighting; in sculpture, however, increased luminosity can reduce the sculptural detail, and may have been compensated via right-side lighting choices within the lighting task.

## Introduction

Visual perception is guided by implicit assumptions in order to make sense of ambiguous stimuli. One of these assumptions is that the direction of light usually comes from above. This phenomenon has been theorized to occur because the earth's universal light source, the sun, is consistently overhead (Ramachandran, 1988) and has been repeatedly demonstrated through experimental study (Berbaum et al., 1983; Sun and Perona, 1998; Mamassian and Goutcher, 2001; Elias and Robinson, 2005; Adams, 2007). In addition to this light-from-above bias, research has also identified a smaller, but reliable leftward lighting bias.

Sun and Perona (1998) demonstrated this lighting bias by having participants immediately indicate when they detected a target sphere among a cluster of distracter spheres. The spheres were laterally-shaded and the light's projected angle varied by trial to determine the strength of the directional preference. Participants exhibited a left-side lighting bias, as target spheres were detected fastest when the lighting direction came from 30 to 60° from the left of vertical center. Lighting bias has also been observed throughout experimental studies using other types of stimuli such as shaded rectangles (Mamassian and Goutcher, 2001) and individual or paired shaded spheres (Elias and Robinson, 2005).

Lighting biases are also evident in visual artworks (Sun and Perona, 1998). Sun and Perona (1998) observed 225 paintings and found that 77% had leftward illumination. Further study reported the leftward bias regardless of the artistic genre (e.g., Renaissance, Impressionist) or the historical era of the painting. Lighting biases have also been reported within advertisements (Thomas et al., 2008) and experimentally demonstrated with photography (Kobayashi et al., 2007). Participants were asked to take photographs either outdoors without using the camera's viewfinder, outdoors and with the viewfinder, and indoors with the viewfinder found a leftward bias only in conditions where the framing composition could be manipulated.

McDine et al. (2011), however, suggested that the artwork examined in these studies did not account for factors that could influence where the vertical light source would be placed, such as the ground or horizon lines, as it would only be logical to provide illumination from above.

To address the confound of equal vertical and lateral lighting placement, the lighting preferences for abstract paintings were chosen as stimuli since the artistic content would not represent any realistic forms or environments (McDine et al., 2011). A virtual art gallery task displayed the stimuli to which participants would place a virtual flashlight on the image in a manner they found to be most aesthetically pleasing. The stimuli were presented individually on the computer screen and the virtual flashlight had a fixed level of luminosity. As predicted, the preferred lighting was placed above more and slightly left of the canvas. This leftward bias could be due to the neurological phenomenon known as pseudoneglect (Thomas et al., 2008; McDine et al., 2011).

Pseudoneglect is a leftward attentional bias theorized to occur due to attentional mechanisms of the right-parietal area of the brain in neurotypical individuals (Jewell and McCourt, 2000). The left-side bias would be facilitated by the contralateral processing of visual information (Riordan-Eva and Cunningham, 2011) and in effect would direct attention to the left-visual field slightly more than the right-visual field (Bultitude and Aimola Davies, 2006). Research assessing pseudoneglect has additionally found that the perception of brightness of shaded-stimuli differ depending on which visual field it is presented to (Bowers and Heilman, 1980; Mattingley et al., 1994; Nicholls and Roberts, 2002; Heber et al., 2010). This has been performed using grayscale judgment tasks which consist of presenting two vertically-aligned rectangles with equal, but mirrored shading gradients beginning from the left or right; participants' task is to then judge which rectangle they perceive to be the darkest (Mattingley et al., 1994). Perceptual bias is indicated if the direction of the rectangle's shading (right or leftward) is chosen to be darker than the other rectangle. This method for assessing pseudoneglect has since been utilized in lighting bias studies to determine the relationship between pseudoneglect and lighting preference (McDine et al., 2011). The present study will also utilize this approach to examine lighting bias from an aesthetic stand point and will do so from a novel perspective.

The previous research on lighting bias and aesthetics have addressed several artistic mediums (e.g., paintings, advertisements), but they are all 2-dimensional forms of representation.

Three-dimensional mediums, such as sculpture, differ based on its unique artistic conventions; placing an external light source on a canvas does not change the aesthetic content within the image, whereas illuminating a sculpture can completely alter the shadows and highlights depending on the sculpture's relief (Kepes, 1995). The purpose of the present study is to address this gap in the literature by assessing the prevalence of lighting bias within the 3-dimensional medium of sculpture. In order to address 3-dimensionality, a virtual lighting task will be implemented which would simulate the creation of an art gallery for free-standing sculptures. The task will allow participants to select combinations of vertical, lateral, and dimensional (i.e., front and back) light for the sculptures in a manner that they find to be the most aesthetically pleasing. Based on the previous literature of lighting bias, the overarching hypothesis is that lights oriented from above and from the left will be selected more often than lights in any other location. The grayscale judgment task will also be presented and is predicted that grayscales will be judged to be darker when the gradients begin from the left side than from the right, and will additionally positively correlate to participant's selections on the lighting task.

## Methods

### Participants

Thirty-nine participants (28 females; *M* = 22.59, *SD* = 4.3) were recruited through the University of Saskatchewan psychology participant pool and were compensated with course credit. Thirty-eight participants were right-handed and one was left-handed which was assessed using the Waterloo Handedness Questionnaire-Revised (Elias et al., 1998). Due to previous evidence that suggests native right-to-left readers tend to exhibit weaker pseudoneglect (Chokron and Imbert, 1993), lighting bias (Smith and Elias, 2013), and opposing aesthetic preferences (Nachson et al., 1999; Chokron and De Agostini, 2000) in comparison to left-to-right readers, only native left-to-right readers were included upon data analysis. Additional inclusion criteria were for participants to have normal or corrected to normal vision and to have no previous participation in laterality studies. Ethical approval was granted by the University of Saskatchewan's Psychology Research Ethics Committee.

### Apparatus

The virtual art gallery lighting task was presented on a 24-inch (61-cm) Acer X243W LCD monitor with the brightness set to 100%. Participants were seated central to the screen which was approximately 22 inches (56-cm) away from the participant. The program was played on a desktop computer running Mac OS X 10.8.5. The grayscales task was presented on a different desktop computer in the same room. The monitor was a 17-inch (43-cm) Sceptre Dragon Eye CRT that participants were seated approximately 14-inches (35.5-cm) away from. The computer used the Windows XP Professional software and the screen was set to 70% brightness intensity. The grayscales task was presented on the CRT monitor due to its faster refresh rate for the rapid presentation of the stimuli, and the LCD monitor was used for the art gallery lighting task for its superior color quality.

The virtual art gallery lighting task was developed using Unity, a cross-platform game engine. The 3D sculptures selected for the task were taken from websites providing free and open-source 3D models. Prior to being imported into Unity, the models were normalized (i.e., creating the same file type, scale, and relative positional anchors) using the open-source 3D modeling software Blender. The scripts necessary for the implementation of the first-person character controller, the rotation and changing of the 3D sculptures, as well as the enabling/disabling of specific lights, were written in the C# programming language. Unity provides its own graphics engine, which, in this case, was set to target the OpenGL graphics API utilized by Mac, Windows, and Linux. Unity's graphics engine was responsible for the rendering of all the lights (i.e., 12 spotlights, and one global light source) and meshes (all 3D models and the gallery itself). The created software is also capable of recording each participant's lighting and posing selections. Posing choices were indicated from rotations beginning at 180° and ranging from 0 to 360°.

### Measures

#### Virtual art gallery task

**Figure 1:**
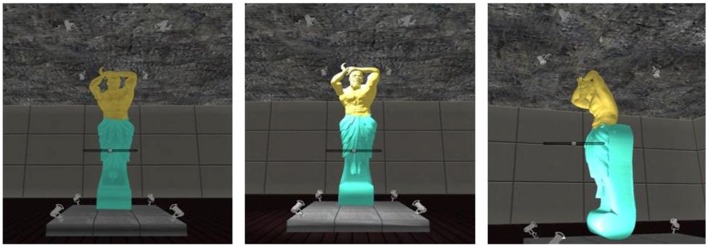
**Example of lighting selections**. The figures from left to right illustrate how the sculpture appears unlit, with the lighting selection from the above-right-front location, and with bottom-right-front lighting viewed from a different spatial perspective. The selected lights were documented based on position (i.e., above-left-front); posing was recorded as rotations starting at 180° and ranging from 0 to 360° (left pose < 175°, right pose > 185°).

The art gallery task allowed participants to choose the lighting placement for free-standing sculptures within an art gallery setting. The program had forty-eight galleries that each contained one sculpture placed in the center of the room in its approximate frontal orientation. The task provided a first-player perspective to create a realistic experience for the participant and to illustrate how the selected lighting illuminated the sculpture from all perspectives. Each gallery was presented with insufficient lighting to which one of eight lighting choices were provided (e.g., left/right, above/below, and front/back) to illuminate the sculpture (see Figure 1). All lighting choices produced equivalent intensities of luminance.

In addition to control of the lighting, the sculpture's pose could also be manipulated to give participants further control over their gallery presentation. The posing rotations were made on a 360° axis that originated at 180°. Sculptures were then scored as “left,” “central,” or “right” posed depending on its original and then selected pose. There were no time constraints given for lighting or posing selections to allow participants to carefully choose their preferences for each sculpture (see Supplementary material).

The content of the sculptural stimuli varied in subject matter, orientation, and color for the purpose of providing variety. For the types of sculptures shown, 15 were characterized as representational (i.e., human figures), six as abstract (defined as the form being its sole identifiable property), and two belonging to non-representational art (no depiction of any realistic form). The overall sizes of the stimuli were quite large in order to maximize the details created by the lighting selected, however, there was some variability between the statures of the sculptures. All of the sculptures were characterized as free-standing or in-the-round, with 18 possessing an erect orientation and six which were recumbent; the discrepancy between the numbers of erect from recumbent sculptures is typical within free-standing sculptures, as most are oriented erect. The color also differed by sculpture (i.e., gold, brass) and was done so during the creation of the lighting task to add variety to the stimuli. Each sculpture (except for one) was one solid color to ensure an equal ratio of color across the artworks. An unequal color balance could have affected lighting choices, as brighter hues in areas on the left or right sides of the sculpture could alter the perception of brightness.

To account for possible confounding factors such as the influence of posing on lighting direction, each sculpture's mirror-orientation was also presented. This resulted in 48 sculptures (24 original, 48 with mirror-reversed position) shown in the task. Each presentation (i.e., original and mirror-reversed positions of sculptures) was presented blockwise with a counterbalanced block order. The design of the galleries were created to be windowless and contain only one sculpture at each time to ensure that lighting choices would not be influenced by any external cues or interactions from other artworks. Each participant's choices were compiled separately from the program until ready for further analysis.

#### Grayscale judgment task (Nicholls et al., 1999)

The grayscale judgment task presented pairs of vertically aligned, horizontal rectangles filled with a shaded-gradient. The gradient for a rectangle within a pair was concentrated to either the left or the right side, to which the other rectangle would display its mirrored equivalent (i.e., left or right concentrated shading). The rectangles were 79 pixels high and varied in length between 320 and 720 pixels with 80 pixel increments. Each width was randomly presented in its original or mirror-reversed position, resulting in a 92-item measure. Participants chose which rectangle was perceived to be darker (e.g., the top or bottom rectangle), to which the direction of attentional bias would be indicated by subtracting the choices of leftward shaded rectangles from the rightward ones.

### Procedure

After providing informed consent, participants were seated directly in front of a computer screen to begin the art gallery lighting task. To illustrate a clear narrative for the purpose of the task, participants were given the following scenario:

“Imagine that you are the director of a prestigious art gallery. There is an upcoming sculpture exhibition that many people will be attending. Your assignment is to light and pose the sculptures in a way that makes the sculpture look the most aesthetically pleasing. The position you are in at the beginning is where the entrance to the gallery is located. Theoretically, you may want the sculptures to be presented in their best way toward the entrance to try to maximize the patron's first reaction to the sculpture.”

The computer's controls for the task were then verbally explained. The task would subsequently begin with the participant's player appearing in the first of forty-eight galleries.

Upon appearing in each gallery, the participant would view the frontal perspective of a sculpture that was placed in the center of the room. They could then experiment with the lighting by clicking with the mouse using their dominant hand on one of the eight lights oriented around the sculpture—only one light could be activated at one time, however. The posing direction of the sculptures could also be manipulated and was done so by clicking on a sliding bar that appeared in the center of the screen. Participants could then travel around the gallery to view how their lighting choices interacted with the posed sculpture by using the arrow keys to move and by dragging the mouse to the sides of the screen to change the viewer's perspective; participants were instructed to use their index finger for the “up” arrow and middle finger for the “right” arrow with their right hand, and their index finger for the “down” arrow and middle finger for the “left” arrow with their left hand. Once the participant had made their final selection, they would to press the “]” key which would take them to the next gallery. The same procedure would be repeated throughout all forty-eight galleries.

Upon completion of the lighting task, the grayscale judgment task was presented on a computer to which participants were instructed to choose which grayscale (i.e., top or bottom) they perceived to be darker. Participants would use their preferred hand to press keys labeled as “top” and “bottom” on the keyboard; the labeled keys were oriented one above the other. The program provided the instructions for the task as well as ten practice trials before the 92 recorded trials. The study finished by participants completing a paper copy of the Waterloo Handedness and Footedness Questionnaire-Revised. The study took approximately 45 min to complete.

## Results

### Lighting bias analyses

**Figure 2:**
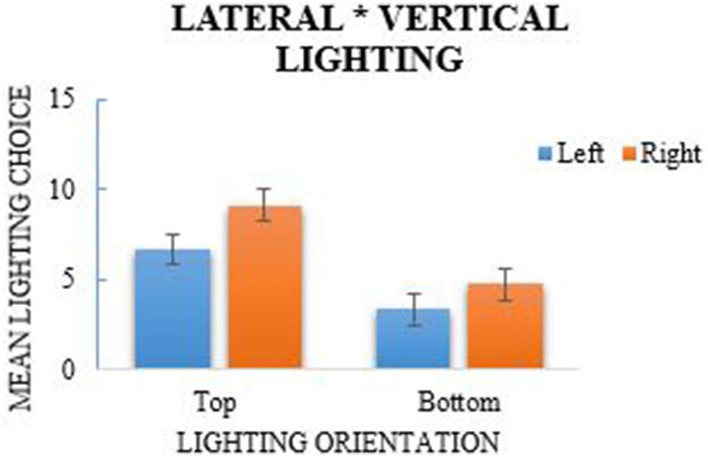
**Main effect of left/right lighting, *F* = 45.5, *p* < 0.001, and non-significant interaction of lateral and vertical lighting choices**. More right-side lights were chosen than left-side lights and more lights were selected from the top than the bottom. Error-bars are 95% Confidence Intervals.

**Figure 3:**
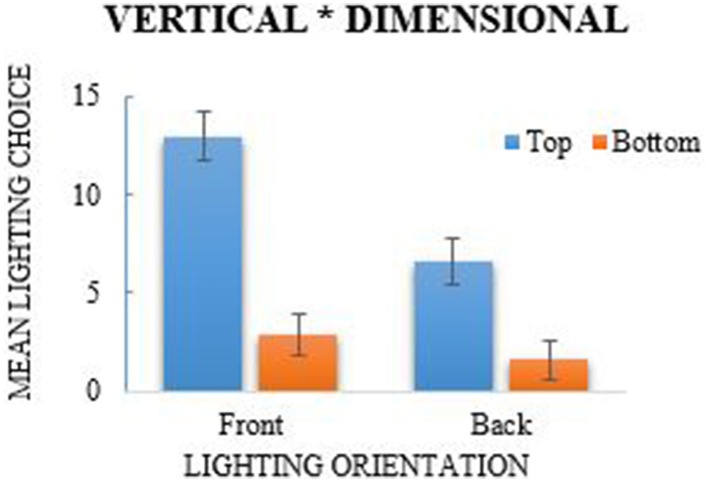
**Main effect of top/bottom lighting, *F* = 25.51, *p* < 0.001, and interaction of vertical and dimensional lighting choices**. More top-lights were chosen in comparison to bottom lights and top-lights were chosen significantly more from across the front and back dimensions. Error bars are 95% Confidence Intervals.

**Figure 4:**
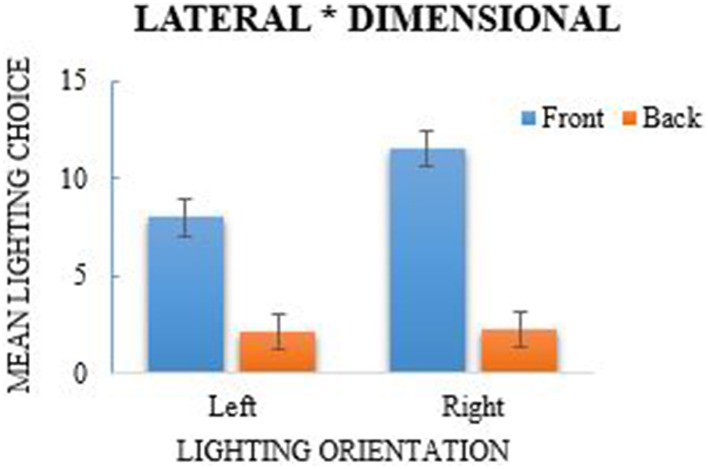
**Main effect of front/back lighting, *F* = 21, *p* < 0.001, and interaction of lateral and dimensional lighting choices**. More lights were selected from the front than from the back and front-lights were chosen significantly more across left and right choices. Error bars are 95% Confidence Intervals.

Each participant's lighting choices in the lighting task were calculated to indicate the frequency that they chose each lighting position (see Table 1). Eight variables were computed using the lighting frequencies to represent each lighting position and was subsequently analyzed using a 2 (left/right lights) × 2 (top/bottom lights) × 2 (front/back lights) repeated-measures ANOVA. The analysis revealed a main effect of lateral (left/right) lighting, *F*_(1, 38)_ = 45.5, *p* < 0.001, η^2^_*p*_ = 0.545, where more right-side lights were selected (*M* = 6.936, *SE* = 0.187) in comparison to left-side lighting choices (*M* = 5.045, *SE* = 0.187; see Figure 2); a Shapiro–Wilk test of normality indicated that lateral lighting was normally distributed (*p* = 0.226). A main effect of vertical (top/bottom) lighting was also observed, *F*_(1, 38)_ = 25.51, *p* < 0.001, η^2^_*p*_ = 0.81, where more selections were made for top lights (*M* = 7.91, *SE* = 0.285) in comparison to bottom lights (*M* = 4.071, *SE* = 0.285; see Figure 3). Vertical selections were not normally distributed as determined by the Shapiro–Wilk test, *p* < 0.001. A chi-square goodness-of-fit was used to address the violation of normality which also reported a significant difference between top and bottom lighting choices, χ^2^_(1)_ = 95.718, *p* < 0.001. A main effect for front and back lighting was also present, *F*_(1, 38)_ = 161.84, *p* < 0.001, η^2^_*p*_ = 0.401, in which front lights (*M* = 9.769, *SE* = 0.297) were chosen more frequently than back lights (*M* = 2.212, *SE* = 0.297; see Figure 4). This was congruent with the results of the chi-square test, χ^2^_(1)_ = 371.436, *p* < 0.001, which was computed as a consequence from the significance of the Shapiro–Wilk test (*p* < 0.001).

**Table 1:** **Frequency and percentage of lighting selections**.

**Lighting position**	**Frequency**	**Percent**
Top-Front-Left	419	22.4
Top-Front-Right	593	31.7
Bottom-Front-Left	204	10.9
Bottom-Front-Right	310	16.6
Top-Back-Left	103	5.5
Top-Back-Right	120	6.4
Bottom-Back-Left	62	3.3
Bottom-Back-Right	61	3.2
Total	1872	100.0

The repeated-measures ANOVA also revealed a significant interaction between lateral and dimensional lighting, *F*_(1, 38)_ = 14.44, *p* = 0.001, η^2^_*p*_ = 0.055 (see Figure 4). *Post-hoc* analyses using paired-sample *t*-tests revealed that lights were chosen significantly more from the front across left and right choices, *t*_(38)_ = 16.57, *p* < 0.001, Cohen's *d*_*av*_ = −0.736, whereas it was not significant when examining back lighting choices. The Shapiro–Wilk test for the ANOVA indicated that there was a skewed distribution (*p* < 0.001), though the results of a chi-square analysis also revealed a significant association between lateral and dimensional lighting, χ^2^_(1)_ = 5.63, *p* = 0.018. An additional interaction was reported between vertical and dimensional lighting, *F*_(1, 38)_ = 21, *p* = 0.001, η^2^_*p*_ = 0.356 (see Figure 3). Normality, however, was violated, and a chi-square test further indicated that the association was not significant, χ^2^_(1)_ = 0.353, *p* = 0.552. There was no significant interaction for lateral and vertical lighting, *F*_(1, 38)_ = 2.22, *p* = 0.144, η^2^_*p*_ = 0.275 (see Figure 2), or for the three-way interaction between lateral, vertical, and dimensional lighting, *F*_(1, 38)_ = 0.97, *p* = 0.331, η^2^_*p*_ = 0.0249.

### Posing and lighting bias analyses

A paired-sample *t*-test was used to compare the lighting selections for sculptures presented in their original orientation (block 1) with the selections from their respective mirror-reversed orientation (block 2); this was to address if the direction of illumination was chosen dependent on the pose of the sculpture. The analysis reported no significant difference, *t*_(38)_ = 0.74, *p* = 0.47, Cohen's *d*_*z*_ = 0.118, two-tailed, indicating that there was no change in lighting choices based on the orientation of the sculpture (i.e., original or mirror-reversed). In order to examine the relation between the overall lateral lights and posing chosen, a chi-square analysis was used. Lighting was coded as “−1” for left-lighting and “+1” for right-lighting choices, and poses were scored as “-1” for left-poses and “+1” for right-poses; central-poses signified no bias and were excluded from the analysis. The comparison was not significantly different, χ^2^_(1)_ = 0.57, *p* = 0.45, suggesting no association between the direction of lighting chosen and the sculpture's posing direction.

### Grayscale and lighting bias analyses

**Figure 5:**
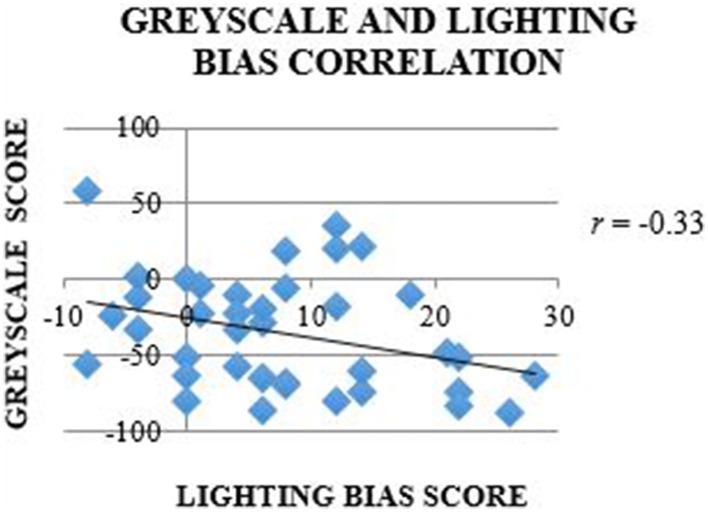
**Correlation between grayscale task and lighting bias scores**. The purpose of the correlation was to identify the association between the directional bias on the grayscales task and for lateral lighting placement in the lighting task. The trend-line illustrates that as the perceived brightness of grayscales was biased to the left (left noted by negative scores), preference increased for lighting from the right for the lighting task (right noted by positive scores).

A one-sample *t*-test (compared to 0) was used to analyze bias scores on the grayscale task. The results indicated a significant leftward bias, *t*_(38)_ = −5.92, *p* < 0.001, Cohen's *d*_*av*_ = −1.921, one-tailed. A bivariate Pearson's correlation was used to examine the relation between performance on the grayscale task and the lateral preference in the lighting task. Left grayscale and lighting task choices were denoted as “−1,” and right-choices as “+1”; the range of scores for the grayscale and lighting task was −92 – 92 and −42 – 42, respectively. A bivariate Pearson's correlation reported the relationship between lighting bias and grayscale judgment scores to be negatively correlated, *r*_(38)_ = −0.33, *p* = 0.042, 95% CI [−0.344 – 0.285] (see Figure 5).

## Discussion

The results of the present study suggested a preference for sculptures to be lit from above, to the right, and in front of the sculpture when in a simulated art gallery setting. The preference for light illumination from above is not surprising given that it has been consistently demonstrated throughout lighting bias studies (Berbaum et al., 1983; Sun and Perona, 1998; Mamassian and Goutcher, 2001; Elias and Robinson, 2005; Adams, 2007), though the discovery of the lateral preference was much more unexpected. Previous research assessing lighting bias and aesthetics has demonstrated a preference for 2-dimensional mediums to be lit slightly from the left (Sun and Perona, 1998; Kobayashi et al., 2007; Thomas et al., 2008; McDine et al., 2011). The present study, however, evidenced a rightward lighting bias when addressing 3-dimensional stimuli which was not in line with the predicted leftward lighting bias. This finding is novel to lighting bias research examining artwork, though the nature of the 3-dimensional medium under study as well as the measure of the lighting task may be directing this opposing bias. In order to interpret the rightward lighting bias, the artistic conventions for lighting sculpture will be considered as well as findings on the recent lighting perception phenomenon of “subjective equality of lighting.”

The theory of subjective lighting equality states that when an object is lit from the left or right under the same lighting intensity, the object would be perceived to be more heavily illuminated from the left-side than from the right (McCourt et al., 2013); the perception of brightness would be biased to the left in light of pseudoneglect, as research on lateral biases have reported that objects tend to be perceived as brighter or darker when presented to the left-visual field (Mattingley et al., 1994, 2004; Nicholls et al., 1999; Nicholls and Roberts, 2002). Support for this theory was provided by McCourt et al. (2013) by presenting participants with two arrays of nine 3-dimensional cubes that varied in pose. One set of cubes, labeled as the matching arrays, varied by one of four lighting directions (e.g., above-left, above-right, below-left, and below-right) and one of fifteen lighting intensities. The second array, labeled as the reference array, was lit only from the above-left position and with a consistent moderate light intensity. In each trial, one condition of matching arrays was presented beside the reference array, to which participants would indicate which one was perceived to be the most intensely lit. The results indicated that cube arrays lit from the left were rated as more intensely lit than from the right. These findings in conjunction with the artistic conventions of lighting sculptural artworks will support the interpretation for the opposing lighting preference reported in the present study.

Based on the theory of subjective lighting equality, it would be assumed that with all lighting choices being equal, sculptures that were lit from the left side would be perceived to be more heavily lit than when lit from the right. This element is significant to recognize, as the goal when lighting sculptures is not to display the piece with the most light, but in a manner that complements the aesthetic details such as the contour, color, and relief (Kepes, 1995). One efficient way of doing this is by ensuring that the lighting is not exceedingly bright, otherwise the features and color of the sculpture can appear to be over-exposed (Zelanski and Fisher, 2006). Because the lighting was of equal intensity for all of the lights in the lighting task, left lights would theoretically decrease the aesthetic features of the sculpture which may have led participants to compensate by choosing lights situated from the right. This phenomenon, however, may be an occurrence that is only witnessed within 3-dimensional stimuli, as the paintings lit with the “virtual flashlight” in the study by McDine et al. (2011) also had a consistent intensity of light, yet evidenced a leftward lighting bias. The interesting factor here is that how the participants are lighting the artwork appears to be related to the medium's dimensionality. Further study should be conducted based on the current study to remedy whether right-side lighting choices were in fact a result of subjective lighting equality as well as actual lighting preference when the light intensity could be manipulated.

In addition to the right-side lighting bias addressed in the primary hypothesis, the secondary hypothesis also provided some findings of interest. Bias on the grayscale task was predicted to be positively correlated with participants' lighting bias on the lighting task. However, the direction of bias on the grayscales task was *negatively* correlated with the lighting bias scores. A positive correlation was predicted because it would suggest that the attentional mechanisms guiding pseudoneglect (measured by performance on the grayscales task) could be influencing the predicted leftward lighting bias (measured by preferences within the lighting task). This was constructed in light of previous research by McDine et al. (2011) who utilized this analysis between their grayscales task and lighting task for 2-dimentional artwork; however, there was no significant correlation reported in their study. The negative correlation between the tasks in the present study cannot imply that the directionality between pseudoneglect and lighting bias are not related, as the explanation of subjective lighting equality for the rightward bias is also a product of pseudoneglect. Limitations of the lighting task also do not allow for a conclusive report between the relationship of directional bias and the tasks.

Limitations presented in the present study arise from several features from the lighting task. A problematic detail is that the scenario read to participants was referred to in future tense (e.g., “There is an upcoming sculpture exhibition… ”). Left-to-right readers (i.e., the study's target participants) tend to mentally construct the future to belong to the right and the past to the left, and has been demonstrated that future and past-tense language can facilitate the direction of spatial attention (Santiago et al., 2007; Oliveri et al., 2009; Bonato et al., 2012). Further study should resolve this by presenting the scenario's instructions in present-tense by simply asking “position and light the sculptures in a manner that you find most aesthetically pleasing.” The lighting task should have also reconsidered the “]” and arrow keys used to travel around the gallery, as research has suggested that symbols implying spatial direction may unconsciously direct one's orientation of attention (Chica et al., 2014). Although the keys in the present study were not used to manipulate the posing or lighting of the sculpture, its functions could have alternatively been placed on keys with no implied directionality while maintaining a vertically and horizontally symmetrical position (e.g., keys H, I, X, O). Lastly, the brief instructions located on the bottom screen of the lighting task should have been removed, as its left-flushed presentation could have biased attention rightward. Previous research has reported that attention tends to shift or “repel” from the location of an object in relation to its line edge to the lateral side of the object (Chieffi et al., 2008, 2012) which demonstrates how the left-flushed text could have also contributed to a right-side lighting preference.

An additional explanation for the rightward bias could be due to processes involved in thematic-role assignment within images. Chatterjee et al. (1995) found that participants tended to illustrate the agent on the left side of an image and the subject on the right side, regardless of the visual content. Due to the active nature of the first-player control provided in the virtual lighting task, participants may have perceived themselves as the agent on the screen and the light source as the subject, therefore facilitating more right-side lighting selections. However, a first-player perspective was similarly given by McDine et al. (2011) when participants were asked to virtually light abstract paintings, yet a left-ward bias was evidenced, parallel to previous studies assessing artwork and lighting biases. The potential effect of perceived agency could be unique to 3-dimensional environments and should be further examined given the rightward lighting preference.

In addition to addressing the discussed limitations, further research should be performed to explain the findings of the present study and to broaden the limited knowledge of lighting bias for 3-dimensional stimuli. If lighting preference remained the construct of interest, the lighting task could be redesigned to allow manipulation of the intensity of illumination; this would better indicate which lights participants aesthetically preferred instead of making the sculpture appear more equally lit. To examine if the rightward preference in the present study was due to subjective lighting equality, paired images of sculptures lit from the left and right in the lighting task could be presented to which participants would determine which sculpture appears to be most equally lit; this methodology is similar to previous research addressing the perceived convexity of left or right-lit spheres (Elias and Robinson, 2005). This task may indicate if lights from the left were perceived to light the sculpture more intensely than right-side lights. Further study could also consider lighting preferences for various types of sculptures (e.g., representational and non-representational, in-the-round and suspended) or non-artistic objects to assess if the direction or presence of preference is dependent on the form of stimuli. Different populations could also be assessed (e.g., left-to-right and right-to-left native readers, artists and non-artists) to explore if culture influences the strength or directionality of lateral bias.

The purpose of the present study was to address the gap in the literature on lighting bias within artwork, as previous research had focused primarily on 2-dimensional mediums of art. The medium of sculpture was chosen as 3-dimensional stimuli and was anticipated that individuals would exhibit a leftward lighting bias when lighting pieces of sculpture, comparable to studies assessing 2-dimensional artwork. The findings indicated the directional bias to in fact be rightward, though previously unknown considerations should have been taken into account given the nature of 3-dimensional stimuli. The results of the study are novel to lighting bias research and will contribute to a greater understanding of the effect that aesthetic lighting preference has on humans' affective responses.

## Author contributions

JS, LE designed research; BW, AS created art gallery lighting task; JS performed research; JS analyzed data; and JS, LE wrote the manuscript.

### Conflict of interest statement

The authors declare that the research was conducted in the absence of any commercial or financial relationships that could be construed as a potential conflict of interest.
